# Medical News and Bulletin

**Published:** 1843-12-23

**Authors:** 


### MEDICAL NEWS AND BULLETIN.

### QUACKERY.

It is curious to observe the circumstances which make people the prey of quacks. There must be first a strong desire for some apparent or real good—health for the sick, bread for the hungry, rest for the overworked, employment and pay for the unemployed. There must be a belief in our own powers to attain these advantages if the proper means were brought within our reach, and a further belief in our power to discriminate the persons and the methods most likely to secure them. This belief in our own sagacity in choosing the best means of relief is the predominant quality in the dupes of quackery ; it produces a morbid state of the elective function, which, like other functional disturbances, if long continued is apt to induce organic disease—and a very troublesome disease it is to the sufferer and those about him. A few weeks ago we met with Father Matthew in a swamp in Bermondsey. If ever dram-drinking were excusable, it was in such a place. But the ruddy good-humoured apostle of temperance preached away *de bon cœur* to fit audience, though few. As one batch of dram-drinkers after another took the pledge, it was impossible not to wish they might keep it. But just behind the scaffold of the water-drinkers was a gallant captain, well known to all frequenters of public meetings, who was holding forth on the virtues of his inhaler, and promulgating his very remarkable theories of disease and political economy. In substance they are these: that the nerves are hollow tubes; that there is no such thing as a solid in the body ; that the great *fomes mali*, the exciting cause of all modern maladies, is white vitriol prepared from human and other bones, for the bakers and brewers, and put into the beer by the orders of government, to brutalize the people, and render necessary the employment of soldiers and policemen. Either in spite of, or in consequence of this instructive lecture, many persons cheerfully underwent the process of inhaling the smoke of a wax candle, and listened with much attention to the advice of the lecturer ; and a young man who begged them not to believe in the sorry delusions of a well-known lunatic, narrowly escaped rough handling, being denounced as a spy and emissary of the doctor.

Now surely the term "debility in action," which has been ingeniously employed to express a morbid state of the bodily functions, is no less applicable to the mental states of these patients. Their elective functions, exceedingly feebly performed in general, were excited by the doctrines and the arguments of te-totallers who had come amongst them, and so, with full confidence in the sagacity with which they had chosen a drinking creed, they proceeded forthwith to choose their medical adviser as above described. The Captain, by the bye, shewed much shrewdness and frankness in saying: "I always follow Paddy Matthew, for he brings out the sick, the lame, and the lazy."

Yet what in these poor people seemed so incredibly silly, that the whole scene was a kind of anachronism in the nineteenth century, a *tableau-moyen agiste* revived in the metropolis of England, may be found every day among the wealthiest of our countrymen, as those can testify who have to deal with the sick, the lame, and the lazy in high places.

About the same time we met with a highly intelligent peer, who consults the most eminent men in the capital for form's sake, and without the slightest belief in their powers of relieving him, but who was expecting with impatience the arrival of a doctor from Vienna, whom he intended to retain in England, at a liberal salary, while superintending a course of a secret remedy for gout, which he determined to undergo.

It is not one of the least evils of quackery, that its pretensions are not always wholly unfounded ; nay, the resources of legitimate medicine are sometimes recruited from its ranks. The London Pharmacopœia retains the paste of Ward, and the powder of Dover, who flourished in the reign of Queen Anne. The men were of unequal merit, their nostrums were alike valuable, and in the lines of Pope their names are embalmed together.

> "See Ward by battered beaus invited over,
> And desperate misery lay hold on Dover."

*Lond. Med. Gaz.* Oct. 27, 1843.

### THE REAL MERITS OF HYDROPATHY.

There is much in the general regimen pursued, or said to be pursued, in hydropathic establishments, the advantage of which the intelligent medical practitioner will assent to, and the practice of which he is in the constant habit of recommending. The regulated diet, regular hours, residence in a pure atmosphere, exercise in the open air, freedom from harassing business, and constant employment, without overstraining of the mental faculties, added to good water as a beverage, instead of acescent and ill-fermented, or too potent fermented liquors, are in themselves sufficient to account for nearly all the benefit which is experienced by such as, in good faith, submit themselves to the treatment enjoined. They are precisely the same dietetic regulations and general regimen which are recommended by every intelligent physician, but which, deficient in the imposing air of a *system*, and appealing only to the reason, instead of playing on the credulity of the patient, are more frequently disregarded than observed, alike to the detriment of the patient, and disappointment of the best directed efforts of his medical adviser.—*Prov. Med. Jour.* Oct. 7, 1843.

### USE OF TEA.

A French Journalist thus learnedly writes: "The difference in the vital instincts of different nations is remarkably exhibited by the use of this beverage (tea;) for who, pray, are they which consume by far the greatest amount of it? Certainly neither the French nor the Italians, not yet the Spaniards; but the English and the Dutch—two nations that are perpetually immersed in a cold heavy and damp atmosphere, and whose inhabitants are soft and flabby in flesh, and dull and phlegmatic in character. (What say you, John Bull, to your portrait?)

(Our wise neighbour writes as if the English and Dutch were the only people who make much use of tea. Not to mention the Americans, may we ask, do the Chinese, and all the Tartar tribes belong to the same category in point of national temperament? The would-be reasoning of the writer should, one might have thought, have led him to the very opposite conclusion ; for if tea acts by being a stimulant, surely coffee is a much greater one ; and therefore it should have suited a phlegmatic people best.)—*Med. Chir. Rev.*

